# Aggravation of cold-induced injury in Vero-B4 cells by RPMI 1640 medium – Identification of the responsible medium components

**DOI:** 10.1186/1472-6750-12-73

**Published:** 2012-10-10

**Authors:** Gesine Pless-Petig, Martin Metzenmacher, Tobias R Türk, Ursula Rauen

**Affiliations:** 1 Institut für Physiologische Chemie, Universitätsklinikum Essen, Universität Duisburg-Essen, Hufelandstr. 55, 45122, Essen, Germany; 2 Klinik für Nephrologie, Universitätsklinikum Essen, Universität Duisburg-Essen, Hufelandstr. 55, 45122, Essen, Germany; 3 Present address: Medizinische Klinik 4 - Nephrologie und Hypertensiologie, Universität Erlangen, Friedrich-Alexander-Universität Erlangen-Nürnberg, Ulmenweg 18, 91054, Erlangen, Germany

**Keywords:** Cell pausing, Cold storage, Iron chelator, Calcium, Phosphate, Preservation, Hypothermia

## Abstract

**Background:**

In modern biotechnology, there is a need for pausing cell lines by cold storage to adapt large-scale cell cultures to the variable demand for their products. We compared various cell culture media/solutions for cold storage of Vero-B4 kidney cells, a cell line widely used in biotechnology.

**Results:**

Cold storage in RPMI 1640 medium, a recommended cell culture medium for Vero-B4 cells, surprisingly, strongly enhanced cold-induced cell injury in these cells in comparison to cold storage in Krebs-Henseleit buffer or other cell culture media (DMEM, L-15 and M199). Manufacturer, batch, medium supplements and the most likely components with concentrations outside the range of the other media/solutions (vitamin B_12_, inositol, biotin, p-aminobenzoic acid) did not cause this aggravation of cold-induced injury in RPMI 1640. However, a modified Krebs-Henseleit buffer with a low calcium concentration (0.42 mM), a high concentration of inorganic phosphate (5.6 mM), and glucose (11.1 mM; i.e. concentrations as in RPMI 1640) evoked a cell injury and loss of metabolic function corresponding to that observed in RPMI 1640. Deferoxamine improved cell survival and preserved metabolic function in modified Krebs-Henseleit buffer as well as in RPMI 1640. Similar Ca^2+^ and phosphate concentrations did not increase cold-induced cell injury in the kidney cell line LLC-PK_1_, porcine aortic endothelial cells or rat hepatocytes. However, more extreme conditions (Ca^2+^ was nominally absent and phosphate concentration raised to 25 mM as in the organ preservation solution University of Wisconsin solution) also increased cold-induced injury in rat hepatocytes and porcine aortic endothelial cells.

**Conclusion:**

These data suggest that the combination of low calcium and high phosphate concentrations in the presence of glucose enhances cold-induced, iron-dependent injury drastically in Vero-B4 cells, and that a tendency for this pathomechanism also exists in other cell types.

## Background

In modern biotechnology and drug design large-scale cell cultures are necessary tools for the production of diverse recombinant proteins, such as Herceptin™, Enbrel™ or vaccines against the influenza virus strains H5N1 and H1N1 like Celvapan™ [1-7]. Cell lines of African green monkey kidney cells (Vero-B4), chinese hamster ovary fibroblasts (CHO) and human embryonic kidney 293 (HEK293) cells are widely used for these purposes [1,2,5,6]. Protein demand and, thus, the demand for cell cultures in protein production fluctuate. Therefore, cold but non-frozen storage of cell lines has been suggested [8,9] to induce an arrest of cell growth by hypothermia, a so-called “pausing” of cells. This would allow a more flexible handling of cell cultures adapted to the demand, e.g. a rapid upscaling of cultures after storage, i.e. keeping cells in “stand-by” storage.

However, hypothermia also induces cell injury [10,11]. This cold-induced cell injury has been shown to be mediated by reactive oxygen species (ROS) formed in an iron-dependent way [10-13] in most cell types. The iron-dependent ROS formation, triggered by an increase in “free”, chelatable iron ions, leads to apoptotic and necrotic cell death via mitochondrial alterations such as an induction of the mitochondrial permeability transition (MPT) [11-17]. This pathway of cold-induced cell injury has been described for various cell types, including human renal proximal tubular cells, rat hepatocytes, rat liver endothelial cells and LLC-PK_1_ kidney cells [16,18].

In addition to this iron-dependent pathway, other changes in cellular ion homeostasis have also been described to contribute to cold-induced cell injury. Classically, a cellular accumulation of sodium due to a reduced Na^+^/K^+^-ATPase activity resulting in cell swelling was thought to cause cold-induced cell injury [19,20]. Newer publications, however, show that sodium plays no role in cold-induced injury in various cell types [17,21,22]. Extracellular chloride, in contrast, has been shown to be involved in cold-induced injury of cultured rat hepatocytes [23].

The use of cell culture medium, in this case DMEM medium, has been suggested for pausing of CHO and HEK293 cells [8,9]. In transplantation medicine, special preservation solutions with often unphysiological, i.e. intracellular ion compositions are used for tissue and organ preservation during extracorporal cold storage, for example University of Wisconsin (UW) solution [19]. However, these preservation solutions also show an inherent toxicity to diverse cell types [23-26]. Krebs-Henseleit buffer (KH) and cell culture media, i.e. media with largely similar “physiological” extracellular ion compositions (in particular with regard to sodium, potassium and chloride), in contrast, yielded a comparatively good cell survival when used for cold storage of rat hepatocytes, rat aortic valves and rat epidermal cells at 4°C [24,27,28]. However, further protection could be observed in the presence of iron chelators [23,24].

Here, we compared KH buffer and cell culture media with and without supplements for cold storage/pausing of Vero-B4 kidney cells. The initial experiments showed an unexpected enhancement of cold-induced cell injury by RPMI 1640 medium, which is the suggested standard medium for Vero-B4 cell culture [29], as compared to KH buffer. Therefore, we performed further experiments to explain this finding and to identify the responsible media component(s) and mechanism, and to elucidate whether this effect is specific to Vero-B4 or kidney cells.

## Results

### Cold-induced cell injury and its aggravation by RPMI 1640

**Figure 1:**
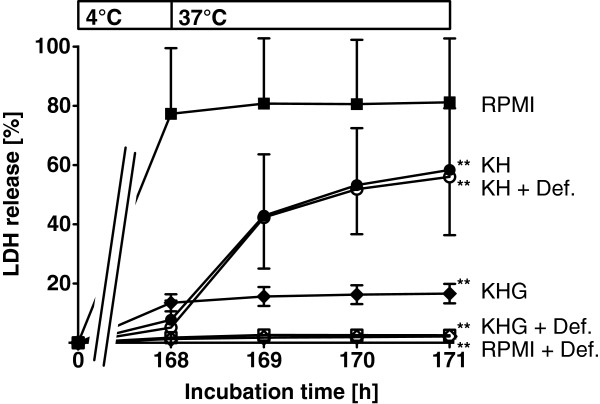
**Cold-induced injury to Vero-B4 cells.** Vero-B4 cells were incubated in RPMI 1640 (RPMI), Krebs-Henseleit buffer (KH) and KH + 11.1 mM D-glucose (KHG) at 4°C for 168 hours and then rewarmed in RPMI 1640 at 37°C for 3 hours. Part of the cells were incubated in the presence of the iron chelator deferoxamine (+ Def.; 1 mM; open symbols). Cell injury was assessed by release of lactate dehydrogenase (LDH; n = 4; ** p < 0.01 vs. RPMI 1640 at 171 h).

Vero-B4 cells stored 168 h at 4°C in Krebs-Henseleit buffer (KH) showed little release of lactate dehydrogenase (LDH) at the end of cold incubation (Figure 1). However, LDH release increased rapidly during rewarming, especially in the first hour. Addition of glucose to KH (KHG) decreased the cell injury during rewarming. Therefore, glucose was added in most of the following experiments to rule out any influence of this rewarming component most likely related to substrate/energy depletion.

In contrast to the results in KH and KHG, very high LDH release was observed at the end of cold incubation of the cells in (complete) RPMI 1640, a cell culture medium containing glucose (Figure 1). Addition of deferoxamine (1 mM) to the media before cold incubation prevented cold-induced cell damage in RPMI 1640 and in KHG but not the rewarming injury in KH without glucose.

Although RPMI 1640 aggravated cell injury in the cold, there was no evidence of a toxicity of RPMI 1640: During warm incubation in RPMI 1640, which is the cell culture medium recommended by the German Collection of Microorganisms and Cell Cultures (DMSZ) for this cell line [29], Vero-B4 cells proliferated normally and showed normal morphology. Using RPMI 1640 from a different company (Sigma instead of Gibco) did not change the amount of cold-induced cell injury seen after cold storage in RPMI 1640 (Table 1).

**Table 1:** Comparison of RPMI 1640 from two different companies

	**LDH release (%)**	
**Conditions of cold incubation**	**RPMI (Gibco)**	**RPMI (Sigma)**
No Inhibitor	92 ± 6	89 ± 4
+ Deferoxamine	01 ± 1	02 ± 0
+ Trifluoperazine + fructose	01 ± 1	01 ± 1
+ Ethanol (solvent control)	90 ± 1	88 ± 4

Vero-B4 cells were incubated in RPMI 1640 purchased from Gibco or Sigma at 4°C for 168 h. The iron chelator deferoxamine (1 mM) or trifluoperazine (20 μM) plus fructose (10 mM), the latter as inhibitor combination of mitochondrial permeability transition [30], were added to part of the incubations during cold storage. Ethanol was used as solvent control for trifluoperazine. Cell injury was assessed by release of lactate dehydrogenase (LDH) after cold incubation (n = 4).

### Role of medium supplements

Comparison of “complete” RPMI 1640 supplemented with foetal bovine serum and penicillin/streptomycin as described in the Methods section, versus “pure” RPMI 1640 showed that the supplements were not responsible for the strong cold-induced injury in RPMI 1640 (Table 2).

**Table 2:** Role of medium supplements

	**LDH release (%)**	
**Solution**	**Without deferoxamine**	**With deferoxamine**
RPMI	66 ± 26	01 ± 1
RPMI without supplements	76 ± 23	02 ± 1

Vero-B4 cells were incubated at 4°C in RPMI 1640 supplemented with fetal bovine serum (10%) and penicillin/streptomycin (25 U ml^-1^/25 μg ml^-1^) or in “pure” RPMI 1640 without supplements for 168 h. The iron chelator deferoxamine (1 mM) was added to part of the incubations. Lactate dehydrogenase (LDH) release was assessed after cold storage (n=4).

### Other cell culture media

**Figure 2:**
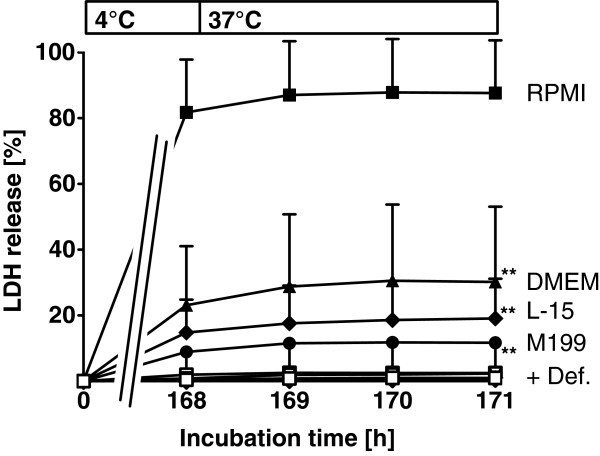
**Cold-induced injury to Vero-B4 cells after cold incubation in different cell culture media.** Vero-B4 cells were incubated in RPMI 1640, DMEM, L-15 and M199 at 4°C for 168 hours and then rewarmed in RPMI 1640 at 37°C for 3 hours. Cell injury was assessed by release of lactate dehydrogenase (LDH). Filled symbols represent media without, open symbols with 1 mM deferoxamine (n = 4; ** p < 0.01 vs. RPMI 1640 at 171 h).

The cell culture media DMEM, L-15 and M199 were compared to RPMI 1640 to evaluate whether enhancement of cold-induced injury is particular for RPMI 1640 or is an effect caused by cell culture media in general. Only cells stored in RPMI 1640 showed a strong cell injury directly after cold incubation (Figure 2). Storage in the other media resulted in far less injury during cold storage; best cell survival was seen in M199. Deferoxamine offered nearly complete protection in all tested media.

### Medium components potentially responsible for the RPMI 1640 effect

As aggravation of cold-induced cell injury was no general effect of cell culture media, we compared the composition of RPMI 1640 with that of the other media and KHG to identify substances or differences in concentrations of substances which could cause the RPMI 1640 effect (Table 3): there were 14 compounds with concentrations in RPMI 1640 outside the range of concentrations in the other media/solutions; of these, the concentrations of Ca^2+^, inorganic phosphate (P_i_) and the components vitamin B_12_, i-inositol, biotin and p-aminobenzoic acid appeared as most likely culprits to cause enhanced cell injury in RPMI 1640. We added vitamin B_12_, i-inositol, biotin and p-aminobenzoic acid to KHG, but none of them proved to be responsible for the enhancing RPMI 1640 effect (Table 4).

**Table 3:** Composition of different cell culture media and Krebs-Henseleit buffer

	**RPMI**	**DMEM**	**L-15**	**M199**	**KHG**
*Na*^*+*^	*139.0*	*157.0*	*145.1*	*144.1*	*143.6*
*K*^*+*^	*5.3*	*5.8*	*5.8*	*5.4*	*5.9*
*Mg*^*2+*^	*0.4*	*0.8*	*1.8*	*0.8*	*1.2*
***Ca***^***2+***^	***0.4***	***1.8***	***1.3***	***1.8***	***2.5***
*Cl*^*-*^	*109.5*	*120.9*	*146.8*	*125.8*	*128.3*
*SO*_*4*_^*2-*^	*0.4*	*0.8*	*0.8*	*0.8*	*1.2*
HCO_3_^ -^	24.0	44.0	14.3	26.2	25.0
***H***_***2***_***PO***_***4***_^***-***^***/HPO***_***4***_^***2-***^	***5.6***	***0.9***	***2.0***	***0.9***	***1.2***
*NO*_*3*_^* -*^	*0.9*	0.7*10^*-3*^	*-*	4*10^*-3*^	*-*
HEPES	-	-	-	-	20.0
Glucose	11.1	25.0	8.3	5.6	11.1
Galactose	-	-	5.0	-	-
Deoxyribose	-	-	-	4*10^-3^	-
Ribose	-	-	-	3*10^-3^	-
***p-Aminobenzoic Acid***	7*10^***-3***^	***-***	***-***	0.4*10^***-3***^	***-***
***Biotin***	0.8*10^***-3***^	***-***	***-***	4*10^***-5***^	***-***
Choline	20*10^-3^	29*10^-3^	10*10^-3^	4*10^-3^	-
Folic Acid	2*10^-3^	9*10^-3^	2*10^-3^	2*10^-5^	-
***i-Inositol***	***0.2***	40*10^***-3***^	10*10^***-3***^	0.3*10^***-3***^	***-***
Niacin	-	-	-	0.2*10^-3^	-
Niacinamide	8*10^-3^	33*10^-3^	10.0*10^-3^	0.2*10^-3^	-
Pantothenate	0.5*10^-3^	8*10^-3^	4*10^-3^	2*10^-5^	-
Pyridoxine	5*10^-3^	20*10^-3^	5*10^-3^	0.1*10 ^-3^	-
Riboflavin	5*10^-4^	1*10^-3^	-	3*10^-5^	-
Thiamine	3*10^-3^	12*10^-3^	3*10^-3^	3*10^-5^	-
***Vitamin B***_***12***_	4*10^***-6***^	***-***	***-***	***-***	***-***
*Glutathione*	3*10^*-3*^	*-*	*-*	2*10^*-3*^	*-*
Pyruvate	-	1.0	5.0	-	-
Phenol red	13*10^-3^	40*10^-3^	30*10^-3^	50*10^-3^	-
Vitamin A	-	-	-	3*10^-5^	-
Calciferol	-	-	-	0.3*10^-3^	-
Menadione	-	-	-	6*10^-5^	-
α-Tocopherol phosphate	-	-	-	2*10^-5^	-
Ascorbic acid	-	-	-	0.3*10^-3^	-
ATP	-	-	-	2*10^-3^	-
AMP	-	-	-	0.6*10^-3^	-
Adenine sulfate	-	-	-	30*10^-3^	-
Guanine	-	-	-	2*10^-3^	-
Hypoxanthine	-	-	-	2*10^-3^	-
Thymine	-	-	-	2*10^-3^	-
Uracil	-	-	-	3*10^-3^	-
Xanthine	-	-	-	2*10^-3^	-
Pyridoxal	-	-	-	1*10^-4^	-
Cholesterol	-	-	-	5*10^-4^	-
L-Arginine	1.2	0.4	2.5	0.3	-
L-Asparagine	0.4	-	1.9	-	-
L-Aspartic acid	0.2	-	-	0. 5	-
L-Cysteine	-	-	1	1*10^-3^	-
Cystine	0.2	0.2	-	0.1	-
L-Glutamic acid	0.1	-	-	0.9	-
L-Glutamine	2.1	4.0	2.1	0.7	-
Glycine	0.1	0.4	2.7	0.7	-
L-Histidine	0.1	0.2	1.6	0.1	-
*L-Hydroxyproline*	*0.2*	*-*	*-*	*0.1*	*-*
L-Isoleucine	0.4	0.8	1.0	0.3	-
L-Leucine	0.4	0.8	1.0	0.9	-
L-Lysine	0.3	0.8	0.6	0.4	-
L-Methionine	0.1	0.2	0.5	0.2	-
L-Phenylalanine	0.1	0.4	0.8	0.3	-
L-Proline	0.2	-	-	0.4	-
L-Serine	0.3	0.4	1.9	0.5	-
L-Threonine	0.2	0.8	2.5	0.5	-
L-Tryptophan	25*10^-3^	0.1	0.1	0.1	-
L-Tyrosine	0.1	0.4	1.7	0.2	-
L-Valine	0.2	0.8	0.9	0.4	-

All concentrations are given in mM. Concentrations of RPMI components printed in italics are outside the range of concentrations in the other media (cell culture media, Krebs-Henseleit buffer with 11.1 mM glucose (KHG)), bold printing highlighting the most likely culprits for the enhanced cold-induced cell injury in RPMI.

**Table 4:** Effects of components potentially responsible for the injurious effect of RPMI 1640

	**LDH release (%)**	
	**Without deferoxamine**	**With deferoxamine**
KHG	12 ± 08	02 ± 1
KHG + i-inositol	14 ± 09	01 ± 0
KHG + biotin	11 ± 06	02 ± 2
KHG + vitamin B_12_	12 ± 09	01 ± 1
KHG + p-aminobenzoic acid	13 ± 10	01 ± 0

Components only present in RPMI 1640 or components of RPMI 1640 with concentrations far outside the range of the concentration in other media were added in identical concentration (inositol, 0.2 mM; biotin, 0.8×10^-3^ mM; vitamin B_12_, 4×10^-6^ mM; p-aminobenzoic acid, 7×10^-3^ mM) to Krebs-Henseleit buffer with 11.1 mM glucose (KHG). Cell injury was assessed by release of lactate dehydrogenase (LDH) at the end of cold incubation (168 h; n = 4).

### Effects of calcium and inorganic phosphate concentrations

**Figure 3:**
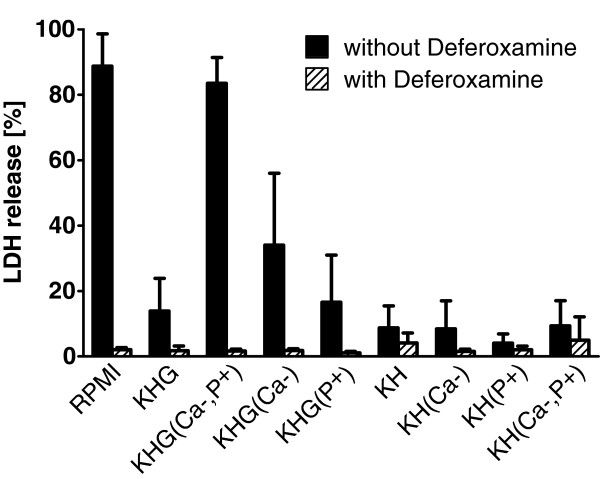
**Influence of modified Krebs-Henseleit buffer on cold-induced injury to Vero-B4 cells.** Vero-B4 cells were stored at 4°C for 168 hours in RPMI 1640, Krebs-Henseleit buffer (KH), modified KH buffer with either 11.1 mM glucose (KHG), low Ca^2+^ concentration (KH(Ca-); 0.42 mM) or high inorganic phosphate concentration (KH(P+); 5.6 mM) alone or in different combinations (KHG(Ca-,P+), KHG(Ca-), KHG(P+), KH(Ca-,P+)). Some cells were stored in the presence of the iron chelator deferoxamine (1 mM, striped bars). Cell injury was assessed by the release of lactate dehydrogenase (LDH; n = 4) directly after cold storage (168 h).

RPMI 1640 contains a lower concentration of Ca^2+^ and a higher concentration of inorganic phosphate than the other media and KHG buffer. Therefore, KHG (with 11.1 mM glucose) was modified to resemble RPMI 1640 medium in these respects (KHG(Ca-,P+); with 0.4 mM Ca^2+^ and 5.6 mM HPO_4_^2-^ as in RPMI 1640). Cold storage in this modified KHG resulted in a similar aggravation of cold-induced cell injury as seen in RPMI 1640 (Figure 3). Further modifications of KH with or without glucose combined with low calcium and/or high phosphate concentrations showed that a combination of all three was necessary to achieve the effect seen in RPMI 1640: The addition of glucose alone (KHG) provoked only a very slight increase in cold-induced cell injury (in comparison to KH), which was similar in the presence of high phosphate (KHG(P+)) and moderately aggravated at low calcium concentrations (KHG(Ca-)). In the absence of glucose, cold storage in all of the modified solutions (KH(Ca-), KH(P+), KH(Ca-,P+)) resulted in little cold-induced injury, similar to that in KH (Figure 3). Only the combination of a reduced Ca^2+^ concentration with an increased concentration of inorganic phosphate in the presence of glucose, KHG(Ca-,P+), evoked a cell injury corresponding to that observed in RPMI 1640. Addition of deferoxamine showed protection in all solutions.

### Metabolic activity

**Figure 4:**
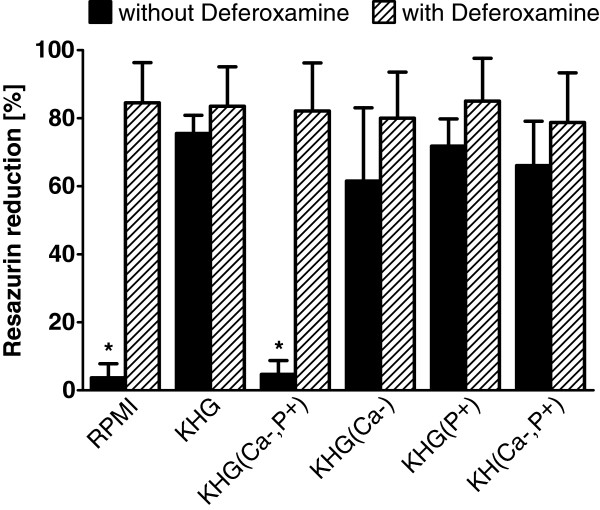
**Reductive metabolism of Vero-B4 cells after cold storage and rewarming.** Vero-B4 cells were incubated at 4°C for 168 hours in RPMI 1640, Krebs-Henseleit (KH) buffer containing glucose (KHG), or modified buffers containing a low Ca^2+^ concentration (KHG(Ca-)), a high inorganic phosphate concentration (KHG(P+)) or different combinations thereof (KHG(Ca-,P+), (KH(Ca-,P+)) and then rewarmed in RPMI 1640 at 37°C for 3 hours. Some cells were incubated at 4°C in the presence of the iron chelator deferoxamine (1 mM; striped bars). The resazurin reduction assay was performed after the rewarming period (171 h of incubation). Resazurin reduction was expressed as percentage of that of non-stored control cells (n = 4; * p < 0.01 vs. KHG).

Assessment of the metabolic activity (resazurin reduction) of Vero-B4 cells incubated for 168 h at 4°C and rewarmed for 3 h confirmed these results: Cells cold stored in RPMI 1640 medium and in the triply modified buffer KHG(Ca-,P+) showed hardly any resazurin reduction (Figure 4). Loss of metabolic activity could be inhibited completely by the addition of deferoxamine during cold incubation. Metabolic activity of cells cold stored in buffer with single or double modifications was only slightly decreased compared to control cells (Figure 4).

### Morphological changes

**Figure 5:**
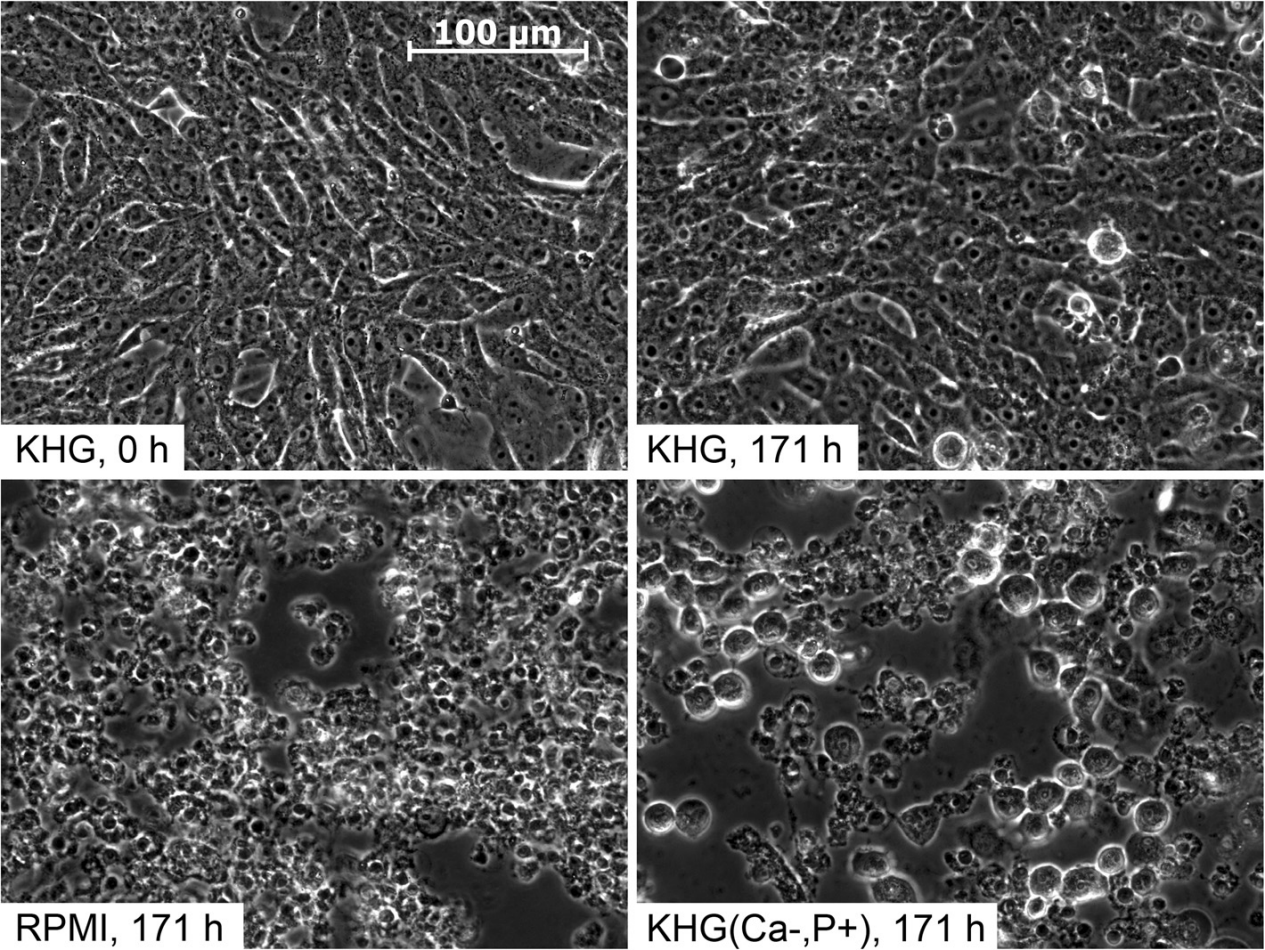
**Morphology of Vero-B4 cells after cold storage/rewarming.** Vero-B4 cells were incubated at 4°C for 168 hours in Krebs-Henseleit buffer with 11.1 mM glucose (KHG), RPMI 1640 (RPMI) and modified KHG buffer containing Ca^2+^ and inorganic phosphate concentrations similar to RPMI 1640 (KHG(Ca-,P+); Ca^2+^ 0.42 mM and inorganic phosphate 5.6 mM) and were then rewarmed in RPMI 1640 at 37°C for 3 hours. Most cell injury was observed in cells cold incubated in RPMI 1640 and KHG(Ca-,P+). The monolayer was disrupted, cells displayed small, dark nuclei and bleb formation occurred. Only cells stored in KHG showed recovery to original morphology.

Assessment of cell morphology also confirmed that the triple combination of glucose, low calcium and high inorganic phosphate is the culprit for the enhancement of cold-induced injury in RPMI 1640: Vero-B4 cells cold-incubated in KHG appeared confluent, and displayed a regular shape after cold storage and rewarming (Figure 5). After cold incubation in RPMI 1640 medium and subsequent rewarming, cells were rounded and partially detached, had small, dark nuclei and pronounced bleb formation could be observed. Similar changes were observed after cold incubation in the triply modified buffer KHG(Ca-,P+) and subsequent rewarming. Addition of deferoxamine to RPMI 1640 and to KHG(Ca-,P+) prevented detachment, nuclear alterations and bleb formation, and a normal monolayer was observed after rewarming (data not shown).

### Evidence for an involvement of the mitochondria

**Figure 6:**
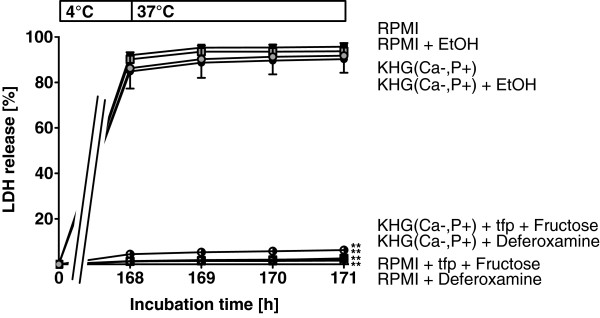
**Blockade of mitochondrial permeability transition (MPT).** Vero-B4 cells were incubated in RPMI 1640 or a modified KH buffer containing glucose, a low Ca^2+^ concentration and a high inorganic phosphate concentration (KHG(Ca-,P+)) at 4°C for 168 hours and then rewarmed in RPMI 1640 at 37°C for 3 hours. Part of the cells were cold incubated in the presence of the iron chelator deferoxamine (1 mM) or of inhibitors of the mitochondrial permeability transition, trifluoperazine (tfp, 20 μM) and fructose (10 mM). A solvent control with ethanol was included (+ EtOH). Cell injury was assessed by release of lactate dehydrogenase directly after cold storage (168 h) and hourly during 3 h of rewarming (LDH; n = 4; ** p < 0.01 vs. RPMI 1640 at 171 h).

Iron-dependent cold-induced cell injury is mediated by a mitochondrial permeability transition (MPT) [13-16]. The MPT inhibitor combination trifluoperazine plus fructose [30] prevented enhancement of cold-induced cell injury in both RPMI 1640 and the triply modified KHG(Ca-,P+) (Figure 6).

### Other cell types

Further experiments were performed with LLC-PK_1_ cells, porcine aortic endothelial cells and rat hepatocytes in an analogous fashion in order to assess whether the enhancement of cold-induced injury by glucose/low calcium/high phosphate is specific to Vero-B4 or kidney cells.

LLC-PK_1_ kidney cells showed slightly higher injury after cold storage in RPMI 1640 and L-15 medium than after cold storage in MEM and M199 and slightly increased injury in the triply modified KH compared to KH, but these effects were neither marked nor significant (Table 5). As described for other cells, the iron chelators deferoxamine (1 mM) and the MPT inhibitor combination trifluoperazine (tfp; 20 μM) plus fructose (10 mM) inhibited cold-induced injury in all solutions.

**Table 5:** Cold-induced cell injury to LLC-PK_**1 **_cells

	**LDH release (%)**	
**Solution**	**Without deferoxamine**	**With deferoxamine**
L15	45 ± 20	1 ± 0
MEM	35 ± 17	1 ± 1
M199	32 ± 17	1 ± 1
RPMI	40 ± 09	2 ± 0
RPMI + EtOH	38 ± 09	
RPMI + tfp + Fructose	09 ± 03	
KH	49 ± 13	
KH(Ca-,P+)	56 ± 15	
KHG	55 ± 13	2 ± 0
KHG(Ca-,P+)	64 ± 14	2 ± 0
KHG(Ca-,P+) + EtOH	63 ± 15	
KHG(Ca-,P+) + tfp + Fructose	08 ± 05	

LLC-PK_1_ cells were incubated at 4°C in RPMI 1640, DMEM, L-15, M199, Krebs-Henseleit buffer (KH), KH + glucose (11.1 mM; KHG) and modified KH buffer containing a low Ca^2+^ concentration and a high inorganic phosphate concentration without (KH(Ca-,P+)) or with glucose (KHG(Ca--,P+)), all with or without the iron chelator deferoxamine (1 mM) for 48 hours. Part of the incubations were performed in the presence of the inhibitors of mitochondrial permeability transition, trifluoperazine (tfp; 20 μM) plus fructose (10 mM). Ethanol was used as solvent control (+ EtOH). Cell injury was assessed by release of lactate dehydrogenase (LDH; n = 4).

**Figure 7:**
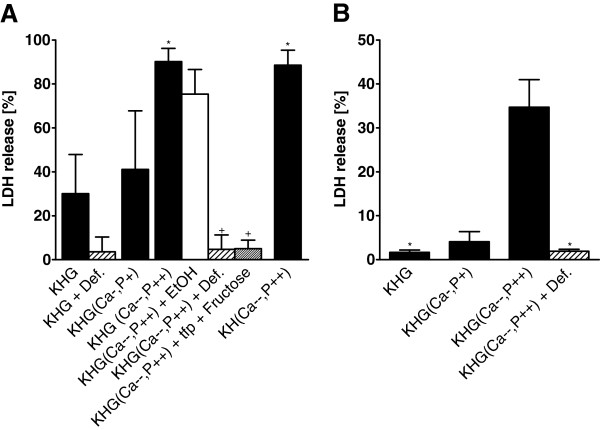
**Influence of modified Krebs-Henseleit buffers on cold-induced injury of hepatocytes and aortic endothelial cells.** Rat hepatocytes (**A**; n = 5) and porcine aortic endothelial cells (**B**; n = 4) were incubated in Krebs-Henseleit buffer with (KHG) or without (KH) glucose and modified Ca^2+^ and P_i_ concentrations for 14 h (rat hepatocytes) or 24 h (endothelial cells) at 4°C. Calcium concentrations in modified buffers were reduced (Ca-, 0.42 mM) or calcium was nominally absent (Ca--), P_i_ concentrations were increased in two steps (P+, 5.6 mM; P++, 25 mM). To part of the incubations, deferoxamine (+ Def.; 1 mM) or trifluoperazine (tfp; 20 μM) plus fructose (10 mM) were added. Ethanol served as solvent control for tfp (+ EtOH). Cell injury directly after cold storage (rat hepatocytes: 14 h, porcine aortic endothelial cells: 24 h) was assessed by release of lactate dehydrogenase (LDH; **A**: * = significantly different from KHG, + = significantly different from KHG(Ca--/P++); **B**: * = significantly different from KHG(Ca--,P++)).

In rat hepatocytes, cold incubation in KH buffer with triple modification (KHG(Ca-,P+)) only slightly increased cell injury after 14 h of cold storage compared to KHG (Figure 7A). However, when P_i_ concentration was increased to 25 mM and calcium was nominally absent (KH(Ca--,P++), as is present in the University of Wisconsin organ preservation solution, cell injury increased severely, regardless of the presence of glucose. However, the enforced triple combination KHG(Ca--,P++) did not only aggravate cold-induced injury but also increased cell injury at 37°C during 14 h incubation (KHG: 10 ± 3%, KHG(Ca--,P++): 41 ± 20%).

In porcine aortic endothelial cells, there was also no increase in cold-induced cell injury in the triply modified KHG(Ca-,P+). However, in the enhanced triple combination KHG(Ca--,P++), cell injury strongly increased during 24 h cold incubation (Figure 7B), but not during 24 h warm incubation (KHG: 4 ± 3%, KHG(Ca--,P++): 16 ± 14%).

## Discussion

Vero-B4 kidney cells showed a massive aggravation of cold-induced cell injury when stored at 4°C in RPMI 1640 cell culture medium, as compared to other cell culture media or KH buffer, and the combination of glucose, low calcium and high phosphate concentrations appeared to account for this phenomenon.

### Mechanisms of cold-induced cell injury

Various cell types display iron-dependent cold-induced cell injury which is triggered by an increase in cytosolic chelatable iron ions [12,31,32]. Iron-dependent ROS formation leads to apoptotic and necrotic cell death via mitochondrial alterations, i.e. induction of the mitochondrial permeability transition (MPT) [13-16]. In all cell types used in this study, cold-induced cell injury could be inhibited by the addition of iron chelators, which indicates that it is mainly iron-dependent. In diverse endothelial cells, the extent of cold-induced lethal cell injury is dependent on the confluence state of the cell cultures, with late confluent cells being particularly prone to injury [33]. On the other hand, subconfluent and early confluent cells are more susceptible to loss of cell-cell and cell-substrate interactions (S. Knoop, U. Rauen, unpublished results). The classical hypothesis of cold-induced cell injury proposes another mechanism based on sodium influx and cell swelling caused by inhibition of the Na^+^/K^+^-ATPase [19,20]. This mechanism could, however, not be verified in adherent rat hepatocytes [22], and also appeared to play little role in the current study (practically no cold-induced injury in the sodium-rich KHG in the presence of deferoxamine, Figures 1, 7, Table 5).

### Enhancement of cold-induced injury by RPMI

RPMI 1640 cell culture medium is the standard culture medium suggested by the German Collection of Microorganisms and Cell Cultures (DSMZ) for culture of Vero-B4 cells [29] and does not show any toxicity at 37°C. Therefore, it was surprising to find that this medium strongly enhanced cold-induced cell injury in these cells (Figure 1). The effect could not be attributed to sodium, as proposed in the classical mechanism, since aggravation did not occur in KH buffer or in other cell culture media with even higher sodium concentrations (see Table 3) but was specific to RPMI. The enhancement was, surprisingly, caused by the triple combination of glucose, low calcium and high phosphate concentrations and was iron dependent, as it was completely inhibited in the presence of iron chelators (Figure 1) and appeared to be mediated by MPT (inhibition by the MPT inhibitor combination tfp/fructose; Figure 6).

### Role of calcium in MPT induction and cold-induced injury

Numerous factors have been discussed to trigger MPT or to sensitize mitochondria to MPT, thus leading to MPT induction either alone or in various combinations [34-38]. Amongst these dozens of factors are increased matrix calcium concentrations, high inorganic phosphate concentrations, decreased mitochondrial membrane potential, oxidation of pyridine nucleotides and of sulfhydryl groups, oxidizing agents and oxidative stress and a mitochondrial matrix pH around 7.4. While accumulation of calcium in the mitochondrial matrix has been and predominantly still is regarded as a prerequisite of MPT induction [35,37], MPT has also been described to occur in the absence of major Ca^2+^ changes, especially when P_i_ is elevated [34,35,39].

It has been shown in various cell types that cytosolic and/or mitochondrial Ca^2+^ concentrations increase during cold ischemia/hypoxia or during subsequent reperfusion/reoxygenation [39-41]. Therefore, most organ preservation solutions contain no or very little Ca^2+^. Protection against anoxia-induced MPT by low extracellular calcium concentrations, associated with a decrease in mitochondrial matrix calcium content, was seen by Pastorino et al. [42]. Also in pure hypothermic injury (without accompanying hypoxia) increases in cytosolic and mitochondrial calcium have been reported and related to cell injury [43-45].

However, Ca^2+^-free incubation aggravated cold-induced cell injury in rat hepatocytes [46-48] and liver endothelial cells [48] and Ca^2+^-free incubation was associated with increased ROS formation at 37°C [49]. In line with this, addition of calcium to clinically used (phosphate-rich) preservation solutions reduced cell damage in rat livers [50,51] and rat aorta [52], and decreased lipid peroxidation [51]. Here, in the presence of glucose, low Ca^2+^ concentrations also increased cold-induced injury in Vero-B4 cells (Figure 3).

### Role of phosphate in MPT induction

Increased concentrations of inorganic phosphate (P_i_) are another well-known trigger for MPT [34,35,37,53]. The deleterious effect of high matrix P_i_ concentrations has been explained by the buffering capacity of P_i_ yielding a matrix pH in favor of MPT [35,37], by the ability of P_i_ to decrease the levels of ADP [37,53], which is supposed to be a potent inhibitor of MPT, or by increasing ROS formation [34,54,55]. To promote MPT, phosphate apparently needs to enter the mitochondrial matrix [35,37,55]. In the present study, increased extracellular phosphate concentrations (5.6 mM) in KHG alone had no influence on cold-induced injury of Vero-B4 cells – only in combination with decreased Ca^2+^ concentrations (0.42 mM) we saw the aggravating effect (Figure 3). This finding is in contrast to the literature, where increased matrix concentrations of calcium are mostly regarded as prerequisite for MPT, although increased P_i_ levels appear to lower the threshold for Ca^2+^, even to physiological levels [34,35,37,39,55]. Potentially, in our setting, enhanced ROS formation during cold storage, likely further increased by low Ca^2+^ concentrations [49], sensitized the mitochondria for phosphate-triggered MPT.

### Role of glucose in enhancement of cold-induced injury

During cold storage, iron-dependent injury in Vero-B4 cells was slightly (KH) or moderately (Ca^2+^- or P_i_-modified KH) enhanced by addition of glucose (Figure 3). Lehnen-Beyel et al. [56] found that in L929 cells, addition of glucose caused an increase in intracellular levels of NADH which enhanced redox-cycling of iron ions and thus aggravated iron-dependent cell injury. Here, the addition of iron chelators inhibited cold-induced injury in all cell types, showing that the injury is also iron-dependent. Since cell lines tend to be highly glycolytic, increased reduction of nicotinamide adenine dinucleotides, i.e. increased availability of NADH, which fosters iron redox-cycling [56], might be the reason for the effect of extracellular glucose in Vero-B4 cells. In hepatocytes, in which no noticeable aggravation of cell injury was seen in the presence of glucose, endogenous glucose from glycogenolysis is likely to be available for metabolism also during incubation in glucose-free KH buffer. However, it should be noted that glucose did not only exhibit injurious features during cold incubation/rewarming but also appeared to be necessary as a substrate for the cells (Figure 1).

### Triple combination

The aggravation of cold-induced cell injury by the triple combination seen here is thus likely to be caused by several interacting mechanisms: during cold storage, iron-dependent ROS formation is further increased due to both, the low Ca^2+^ concentration and simultaneously increased iron redox-cycling fostered by glucose via NADH availability. The increased ROS formation likely sensitizes the mitochondria for MPT. Additionally, MPT is promoted by increased concentrations of inorganic phosphate. Neither of the components alone nor in different combinations of two did approximate the level of cell injury seen for Vero-B4 cells in RPMI during cold incubation – the interaction of all three factors appeared to be necessary for the injurious effect.

### Enhancement not specific for Vero-B4 cells but differences in sensitivity between cell types

The aggravation of cold-induced injury by a combination of glucose, low calcium and high phosphate, although not specific for Vero-B4 cells, seems to be particularly pronounced in this cell type. Porcine aortic endothelial cells also displayed an aggravation of cold-induced injury, but only in more extreme, but still clinically relevant conditions, i.e. the nominal absence of Ca^2+^ and presence of higher phosphate concentrations (0 mM/25 mM as in KHG(Ca--,P++)). In rat hepatocytes, similar phosphate concentrations also induced cell injury, but in these cells, an injurious effect of high phosphate was also seen at 37°C, in line with data described previously [24], and roughly doubled during cold storage (Figure 7).

### Consequences for cell pausing media and organ preservation solutions

Not only RPMI 1640 cell culture medium and organ preservation solutions, but also many well-established buffer solutions (0.05 M phosphate buffer (50 mM phosphate, no calcium), phosphate-buffered saline (12 mM phosphate, no calcium)) display similar characteristics as the modified solutions used here. University of Wisconsin solution [57], which is used for organ preservation in the clinical setting, combines high P_i_ with nominal absence of Ca^2+^ (although in the absence of glucose) in concentrations that are identical with the concentrations we here used in KH(Ca--,PP++) and KHG(Ca--,P++). In Euro Collins solution, the P_i_ concentration is even higher and the solution contains glucose [58]. Considering that hepatocytes and endothelial cells were severely damaged in this environment at 4°C, it should be considered to use phosphate-free/phosphate-poor solutions for cell, tissue and organ preservation. Also, the choice of pausing medium for cell cultures and the solutions used in processing steps performed at lower temperatures should be carefully made, considering that some of the solutions severely aggravate cold-induced cell injury. Addition of iron chelators provides significant protection in many solutions [23,25,32,59-61]. However, with increasing storage time, differences between different base solutions become more distinct even in the presence of iron chelators [59,61]. As iron chelators can cause iron depletion of cells [62,63] and thus interfere with cell proliferation after rewarming, their concentration should be kept at the lowest effective concentration; therefore, enhancing/injurious effects of the base solutions should be minimized. Thus, we suggest using iron chelator-containing solutions based on buffers other than phosphate, such as recently described for cold storage of various cell types [59,64,65].

### Comparison of cell culture media with cold storage solutions

The organ preservation solution UW has been adopted for short-term cold storage of cells with reasonably good results [66-68]. However, for various cell types, UW did not provide better protection than cell culture medium, and cold-induced cell injury in UW could also be greatly reduced by the addition of iron chelators [25,32,59]. In Vero-B4 cells, UW provided better protection than RPMI during one week of cold storage, but the protective effect was lost after longer cold storage periods (B. Akyildiz, U. Rauen, unpublished results). After two weeks of cold storage in UW solution plus three hours of rewarming, LDH release of Vero-B4 cells was about 50% whereas it was less than 10% after cold storage in ChillProtec and ChillProtec Plus, commercially available cell storage solutions. However, the focus of the current study was to understand the surprising RPMI effect, not to compare or further optimize cold storage solutions.

### Limitations of the current study

Limitations of the current study are the relatively short cold storage period, the relatively short follow-up period and the lack of comparison to different cold storage solutions. The storage period of one week and the short follow-up period of 3 h rewarming were chosen to study the disastrous RPMI effect, which is already marked at these time points. However, the short rewarming period does not account for late apoptosis or for proliferative dysfunction of the surviving cells after cold storage. Optimization of cold storage solutions in addition requires longer cold storage periods and proper comparison with the different cold storage solutions available and is currently in progress.

## Conclusion

The aggravation of cold-induced injury to Vero-B4 cells in RPMI 1640 could be attributed to a combination of glucose, low calcium and high phosphate concentrations, which induced cell death most likely via MPT induction. This injury was iron-dependent and could be inhibited by addition of iron chelators. Based on these findings, we suggest low-phosphate storage solutions with iron chelators for cell pausing at 4°C.

## Methods

### Chemicals

RPMI 1640, DMEM and penicillin/streptomycin were obtained from Invitrogen (Darmstadt, Germany), M199 was from Biochrom AG (Berlin, Germany) and deferoxamine mesylate (Desferal) from Novartis Pharma (Nuremberg, Germany). All other chemicals were of analytical grade and obtained either from Sigma Aldrich (Taufkirchen, Germany) or from Merck (Darmstadt, Germany).

### Cell culture

Vero-B4 and LLC-PK_1_ cells were from the German Collection of Microorganisms and Cell Cultures (Deutsche Sammlung von Mikroorganismen und Zellkulturen GmbH; DSMZ). Cells were cultured in 75 cm^2^ culture flasks (Sarstedt, Nümbrecht, Germany) in RPMI 1640 medium supplemented with 10% foetal bovine serum, 2 mM L-glutamine and penicillin/streptomycin (25 U ml^-1^/25 μg ml^-1^) at 37°C in a 100% humidified atmosphere of 5% CO_2_/95% air. Confluent cultures of the cells were split 1:8 and seeded onto 6-well-plates (Sarstedt, Nümbrecht, Germany) for experiments. After three days confluent cell cultures were used for experiments. Primary rat hepatocytes were isolated from male Wistar rats as described previously [11], seeded on 6-well-plates at 10^6^ cells/well in supplemented Leibovitz L-15 cell culture medium [11] and used for experiments 20 h after isolation (approximately 600000 cells/well). Porcine aortic endothelial cells were isolated from porcine aortae as described previously [69], cultured in 25 cm^2^ and, after passaging, in 75 cm^2^ cell culture flasks (Sarstedt, Nümbrecht, Germany) in M199 cell culture medium supplemented with 20% foetal bovine serum, 2 mM L-glutamine, 100 U/ml penicillin and 100 μg/ml streptomycin. First passage cells were split 1:3 on 6-well-plates and cell cultures were used for experiments after 48 h in an early confluent state (at approximately 10^6^ cells/well).

### Experimental procedures

All cells received fresh cell culture medium 20–24 h before the experiments started. The cells were washed three times with Hanks’ Balanced Salt Solution (HBSS, 37°C) at the beginning of the experiment and then covered with cell culture medium, Krebs-Henseleit buffer (KH; NaCl 115 mM, NaHCO_3_ 25 mM, KCl 5.9 mM, MgCl_2_ 1.2 mM, NaH_2_PO_4_ 1.2 mM, Na_2_SO_4_ 1.2 mM, CaCl_2_ 2.5 mM, Hepes 20 mM, pH 7.35) or modified KH (see below) at room temperature.

The following modifications of KH were used in the experiments:

KH(Ca-): low Ca^2+^ concentration (0.42 mM as in RPMI)

KH(Ca--): very low Ca^2+^ concentration, i.e. Ca^2+^ nominally absent

KH(P+): high inorganic phosphate concentration (5.6 mM as in RPMI)

KH(P++): very high phosphate concentrations as in University of Wisconsin (UW) solution (25 mM)

KHG: D-Glucose added in the same concentration as in RPMI 1640 (11.1 mM)

or combinations thereof, for example KH(Ca-,P+), with low calcium and high phosphate concentrations (similar to RPMI), KHG(Ca-,P+) with additional glucose, or KH(Ca--,P++), with very low calcium and very high phosphate concentrations (similar to UW solution). Osmolarity (calculated osmolarity) of modified KH was adjusted by reduction of NaCl.

6-Well-plates were put into airtight vessels that were gassed with 5% CO_2_, 21% O_2_ and 74% N_2_. Vessels were cold stored at 4°C for different time periods dependent on cell type and based on previous experience (Vero-B4 cells 168 h, LLC-PK_1_ cells 48 h, rat hepatocytes 14 h, porcine aortic endothelial cells 24 h). Deferoxamine (1 mM) or trifluoperazine (20 μM) plus fructose (10 mM) were added to some incubations. Inhibitors were only present during cold incubation; ethanol was used as solvent control for trifluoperazine. After cold incubation, part of the cells were washed with cold HBSS, supplied with cold cell culture medium and rewarmed to 37°C in an incubator containing an atmosphere of 5% CO_2_/95% air for 3 h.

### Assays

#### Lactate dehydrogenase release

Extracellular activity of the cytosolic enzyme lactate dehydrogenase (LDH) was measured at the end of cold incubation and during rewarming using a standard enzymatic assay based on pyruvate-dependent NADH oxidation [70]. Residual cellular LDH activity was measured at the end of the incubation after cell lysis with Triton X-100 (1% in HBSS, 30 min). LDH values were corrected for change in volume of incubation medium resulting from repetitive sampling. Released LDH activity was given as a percentage of total LDH activity.

#### Alamar blue (resazurin reduction) assay

Cells not stored cold (control) and cold stored/rewarmed cells were washed carefully with HBSS. Then HBSS containing 10 mM glucose and resazurin at a concentration of 40 μM was added. Cells were incubated in a fluorescence microplate reader (Fluostar OPTIMA, BMG Labtech; Offenburg Germany) at 37°C for 12–15 min (depending on cell type). The fluorescence increase (i.e. reduction of resazurin to resorufin) over time was assessed continuously at λ_exc._= 560 nm and λ_em._= 590 nm. Reduction rate was calculated from the slope of fluorescence increase in the linear range. Reduction rate of cells exposed to hypothermia and rewarming is given as percentage of that of control cells (in which the assay was done at time zero).

### Statistics

All experiments were performed in duplicate and repeated at least three times (see individual figure/table legends). Data are expressed as mean ± standard deviation (SD) unless mentioned otherwise. Two-way ANOVA with Bonferroni multiple comparison as post-hoc tests for parametric data was used to analyze LDH release of Vero-B4 cells. One-way ANOVA with Bonferroni multiple comparison as post-hoc test was used to analyze fluorescence increase and LDH release of the other cell types. Statistical significance level was set at α = 0.05.

## Abbreviations

ROS: Reactive oxygen species; MPT: Mitochondrial permeability transition; UW: University of Wisconsin solution; HBSS: Hanks’ Balanced Salt Solution; KH: Krebs-Henseleit buffer; LDH: Lactate dehydrogenase.

## Competing interests

U. Rauen obtained consulting fees from Dr. Franz Köhler Chemie GmbH, Bensheim, Germany, which holds a patent on a new preservation solution.

## Authors’ contributions

UR designed the study. MM, GPP and TRT performed the experiments and drafted the manuscript. GPP and TRT analyzed the data, GPP and UR prepared the final manuscript. All authors read and approved the final manuscript.
